# Exploring the mediating roles of stigma and perceived social support between health literacy and health care decision-making barriers among patients with cervical cancer

**DOI:** 10.1016/j.apjon.2025.100716

**Published:** 2025-05-12

**Authors:** Hanjiao Kong, Jingzhi Geng, Qiuhui Wang, Jie Zhu, Hong Zhao

**Affiliations:** a Chinese Academy of Medical Sciences, Peking Union Medical College, School of Nursing, Beijing, China; b Department of Gynecological Oncology, Cancer Hospital, Peking Union Medical College, Beijing, China; c Department of Gynecological Oncology, Tsinghua Changgung Hospital, Beijing, China

**Keywords:** Cervical cancer, Health literacy, Healthcare decision-making barriers, Stigma, Perceived social support, Chained mediation effect

## Abstract

**Objective:**

This study aims to investigate the association between health literacy, stigma, and health care decision-making barriers in patients with cervical cancer and identifies the mediating roles of stigma and perceived social support in this relationship.

**Methods:**

A cross-sectional study was conducted using convenience sampling, recruiting 322 hospitalized patients with cervical cancer from three tertiary hospitals in Beijing. The participants completed self-reported questionnaires on health literacy, stigma, perceived social support, and health care decision-making barriers. Chained mediation effects were analyzed using Amos 26.0 software.

**Results:**

37.3% of patients with cervical cancer reported delays in seeking medical care. Health literacy was found to have significant negative associations with stigma (*P* ​< ​0.001) and health care decision-making barriers (*P* ​< ​0.001) and a positive correlation with perceived social support (*P* ​< ​0.001). Health literacy indirectly influenced health care decision-making barriers through three mediating pathways: stigma (effect ​= ​−0.073), perceived social support (effect=−0.086), and a combination of stigma and perceived social support (effect ​= ​−0.041), collectively accounting for 27.5%, 32.3%, and 15.4% of the total effect, respectively. The total indirect effect accounted for 75.1% of the total effect.

**Conclusions:**

The health literacy levels among patients with cervical cancer in China are moderate. Stigma and perceived social support serve as partial mediators in the relationship between health literacy and health care decision-making barriers. Health care providers can reduce decision-making barriers by improving health literacy and indirectly lessen them by addressing stigma and enhancing social support.

## Introduction

Cervical cancer is the fourth most common gynaecological malignancy worldwide, with an estimated 604,000 new cases and 342,000 deaths annually.^1^ The burden of this disease varies significantly by region, with more than 85% of cervical cancer-related deaths occurring in low- and middle-income countries (LMICs).^2^ Despite established screening protocols and preventive strategies, delays in seeking health care remain a critical challenge. A systematic review by Allahqoli^3^ found that 32.7% of patients globally experience a diagnosis delay of three months or more, and the likelihood of advanced-stage in LMICs is 2.3 times higher than in high-income regions. This delay has a significant impact on treatment effectiveness. Every 30-day delay in treatment initiation is linked to a 6.8% decrease in the five-year survival rate of cervical cancer patients.^4^

Delays in healthcare-seeking behaviour continue to be a significant issue.^5^ Patients may be unfamiliar with health care processes, lack trust in medical facilities, and experience emotional distress due to social and familial pressures, leading to delayed medical consultations.^6^ For instance, a study in Jordan found that over half of the participants lacked adequate knowledge of colorectal cancer and its screening procedures, which was associated with higher perceived barriers and lower screening participation rates.^7^ A study by Ma Jun^8^ found that approximately 40.71% of young patients with cervical cancer in China delayed seeking medical care, primarily due to negative health beliefs and insufficient awareness of cervical cancer screening. In India, economic constraints, insufficient knowledge, and social influences are considered key contributors to perceived barriers, resulting in a decrease in healthcare-seeking behaviours.^9^

Health literacy refers to an individual's ability to identify their health information needs, access and evaluate relevant resources, and apply this information to make informed health care decisions.^10^ According to Nutbeam,^11^ higher health literacy enhances patients' understanding of their medical conditions and promotes active participation in health care processes. Ryman^12^ highlighted health literacy as a critical predictor of cancer prevention and management, emphasizing its role in improving treatment adherence, reducing distress, and enhancing quality of life. Patients with cervical cancer often demonstrate limited comprehension of the disease's progression and outcomes, exacerbated by low health literacy, which leads to delayed medical consultations and poorer prognoses.^13^ Zewdie^14^ noted that patients with insufficient health literacy face difficulties in understanding medical information, communicating with health care providers, and navigating complex health care systems, all of which exacerbate perceived barriers to health care and lead to treatment delays or abandonment.

Stigma is a crucial factor that may mediate the relationship between health literacy and health care decision-making barriers. Due to the association of cervical cancer with the female reproductive organs and social taboos, there is a widespread sense of shame among patients with cervical cancer, which often leads to embarrassment and reluctance to seek medical care or discuss symptoms, resulting in delayed diagnosis and treatment. Screening programs are essential for early detection, but studies in Nepal^15^ and Peru^16^ have shown significantly lower screening rates among patients experiencing stigma. The Health Belief Model posits that stigma can amplify perceived barriers, leading to avoidance behaviors and false optimism about self-recovery.^17^

Salehiniya's study explicitly identifies that a lack of social support is a significant barrier for patients with cervical cancer to participate in screening and healthcare-seeking behaviors.^18^ Perceived social support refers to an individual's awareness and belief in the support available from their social network, including emotional, informational, and practical support, which significantly influences patients' mental health and decision-making behaviors.^19^ The main effect model of social support suggests that a strong support system, whether during times of stress or in daily life, positively impacts mental health by providing external resources such as material and informational support. This, in turn, directly promotes physical and psychological well-being, encouraging individuals to actively seek health assistance. Adebola's research further highlights that a lack of social support leads to emotional isolation, information deprivation, and practical difficulties, thereby hindering patients with cervical cancer from engaging in screening behaviors and delaying their healthcare-seeking actions. Specifically, the lack of support from family, friends, and communities hinders patients' access to critical information regarding cervical cancer screening, such as the necessity, methods, and available facilities. Additionally, when patients attempt to attend screenings, they face challenges such as transportation issues, communication barriers, and inadequate financial support, ultimately missing the opportunity for early detection and treatment.^20^ Furthermore, studies have found a correlation between perceived social support and stigma. Patients with lower perceived social support tend to experience higher levels of stigma,^21^ which may further exacerbate their reluctance to seek screening and health care, creating a negative feedback loop.

Although existing literature predominantly focuses on the direct relationship between health literacy and health care decision-making barriers, there is insufficient evidence on how stigma and perceived social support mediate this relationship, particularly in the context of low- and middle-income countries. Previous studies have focused on the direct effects of health literacy, ignoring the interactive roles of stigma and support networks. Therefore, this study aims to explore the mediating effects of stigma and perceived social support in the relationship between health literacy and health care decision-making barriers, providing a theoretical basis for clinical interventions and offering practical guidance to ensure timely medical care for patients with cervical cancer. It is expected that this study will contribute to reducing cervical cancer mortality, improving patient prognosis, and enhancing quality of life.

**Fig. 1:**
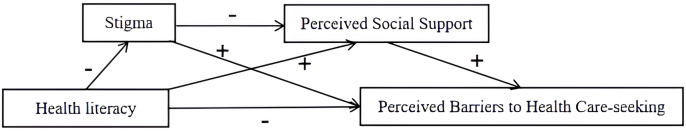
Hypothesized mode.

Therefore, we hypothesize that: health literacy negatively predicts and directly influences perceived barriers to health care decision-making (Hypothesis 1); stigma mediates the relationship between health literacy and health care decision-making barriers (Hypothesis 2); perceived social support mediates the relationship between health literacy and health care decision-making barriers (Hypothesis 3); and perceived social support and stigma act as chained mediators in the relationship between health literacy and health care decision-making barriers (Hypothesis 4). The proposed conceptual framework (Fig. 1) provides strong evidence to support the development of targeted interventions to improve the quality of life and health care decision-making in patients with cervical cancer.

## Methods

### Participants and design

This study is a cross-sectional survey conducted using convenience sampling. From October 2023 to June 2024, patients hospitalized in the gynecologic oncology wards of three tertiary hospitals in Beijing were selected. Inclusion criteria were: (1) diagnosis of cervical cancer; (2) age between 18 and 79 years; (3) awareness of their condition and willingness to sign the informed consent form. Exclusion criteria were: (1) coexisting mental illness or severe organic diseases preventing cooperation; (2) communication or comprehension impairments; (3) coexisting malignancies at other sites.

### Sample size

The sample size was estimated by applying a metric of 5–10 respondents per item in a validated survey to ensure sufficient statistical power.^22^ The study included 24 independent variables, and the sample size was increased by 20% to account for potential invalid questionnaires, resulting in a required minimum sample size of 288.

### Data collection and procedure

All participants were personally invited to complete the survey in a private, quiet room at the hospital without the presence of other members or friends. At the outset, each participant was thoroughly informed about the purpose and methodology of the study, ensuring they understood that they could withdraw from the survey at any time without any impact on their treatment. The researchers distributed the Chinese version of the questionnaire to the participants, who completed it anonymously and independently. During the survey, the researchers were available to assist participants in understanding any confusing items. Participants took approximately 10–15 minutes to complete the questionnaire, which consisted of self-reported questions. After the survey, trained researchers reviewed the responses to ensure all required data were accurately completed. All participants were informed that the collected data would remain confidential and would be used solely for research purposes.

### Measurements

#### Demographic and clinical characteristics

Demographic and clinical characteristics, including age, education, monthly income, occupation, marital status, patient delay, and clinical data such as cervical cancer staging, initial symptoms, patient delay (> 3 months) and comorbidities (e.g., hypertension, diabetes, or dyslipidemia), were collected through self-reports by health care staff or from hospital medical records.

#### Measurement of health literacy

Using the Scale on Health Literacy for Patients with Chronic Disease, developed by Professor Jordan and colleagues from the University of Melbourne, Australia,^23^ The scale consists of four dimensions and 24 items: information acquisition ability (9 items), communication and interaction ability (9 items), willingness to improve health (4 items), and willingness to seek economic support (2 items). Responses were scored using a 5-point Likert scale, with scores ranging from 1 (almost impossible) to 5 (no difficulty) for each item. The total score ranges from 24 to 120. The Cronbach's *α* coefficient in this study was 0.95.

#### Measurement of health care decision-making barriers

Perceived Barriers to Health Care-seeking Decision-Chinese (PBHSD-C), developed by Al-Hassan,^24^ originally included two dimensions: perceived severity of the illness and perceived barriers to seeking medical care, It was later revised and translated into Chinese by Li transforming it into a uni-dimensional scale,^25^ The scale includes 10 items: perceived long waiting times for medical care, reluctance to disturb doctors, feeling embarrassed to seek care immediately when symptoms appear, discomfort in seeking care, facing painful diagnosis and treatment, increased family pressure, restricted social activities, inability to complete work, giving up hobbies, and consulting family members before seeking care. Responses are rated on a 6-point Likert scale, ranging from 1 (strongly disagree) to 6 (strongly agree), for each item. The total score ranges from 10 to 60, with lower scores indicating fewer perceived barriers (10–26), moderate scores indicating medium-level barriers (27–43), and higher scores indicating more significant barriers (44–60). A higher total score indicates more significant health care decision-making barriers. The Cronbach's *α* coefficient in this study was 0.74.

#### Measurement of stigma

We used the Social Impact Scale (SIS), developed by Fife^26^ in 2000, originally designed to measure stigma in cancer and HIV/AIDS patients to assess stigma in this study. The scale consists of four dimensions and 24 items: social exclusion (9 items), internalized shame (5 items), economic insecurity (3 items), and social isolation (7 items). The first two dimensions assess actual stigma, while the latter two assess perceived stigma. The scale uses a 4-point Likert scale, with responses ranging from to scored from 1 (strongly disagree) to 4 (strongly agree). The total score ranges from 24 to 96, with low stigma levels scoring 24–37, moderate stigma levels scoring 38–51, and high stigma levels scoring 52–96. A higher score indicates a higher level of stigma. The Cronbach's *α* coefficient in this study was 0.81.

#### Measurement of perceived social support

The Perceived Social Support Scale (PSSS), developed by Zimet,^27^ assesses individuals' perceived social support across three dimensions: support from friends, family, and others. It consists of 12 items, rated using a 7-point Likert scale, with responses ranging from 1 (strongly disagree) to 7 (strongly agree) for each item. The total score ranges from 12 to 84, with scores between 12 and 36 indicating low perceived support, 37 to 60 indicating moderate support, and 61 to 84 indicating high support. Higher scores reflect better perceived social support. The Cronbach's *α* coefficient in this study was 0.86.

### Data analysis

Data were entered into EpiData software and analyzed via SPSS26.0 and Amos 24.0 software. Participant characteristics, including age, marital status, monthly income, family history, fertility intention, time since diagnosis and types of cancer, were described using frequency and percentage.

Health literacy, perceived social support, stigma, and health care decision-making barriers were described using mean ​± ​standard deviation (M ​± ​SD). Independent samples *t*-tests or one-way analysis of variance (ANOVA) were conducted to test differences in health literacy scores among patients with different characteristics. Pearson correlation analysis was used to examine the relationships between health literacy, perceived social support, stigma, and health care decision-making barriers. Structural equation modelling (SEM) was employed to assess model fit, with health literacy as the independent variable, perceived social support and stigma as mediators, and health care decision-making barriers as the dependent variable, and the model fit was evaluated using χ^2^/df, CFI, TLI, RMSEA, and SRMR indicators. If χ^2^/df ​< ​5, CFI > 0.9, TLI > 0.9, RMSEA < 0.08, and SRMR < 0.08, the model was considered to fit the data well.

A non-parametric bootstrap method was applied to test the mediating effects between health literacy, perceived social support, stigma, and health care decision-making barriers. A two-tailed *P* value of < 0.05 was regarded as statistically significant.

All residual variances (e1-e14) associated with observed variables were included in the structural equation model to account for unexplained variances. These residuals represent measurement errors and unexplained variance in the observed variables. Although not fully displayed in the diagram for clarity, they were considered in the model estimation and evaluated for statistical significance.

## Results

### Common method bias test

Harman's single-factor test was used to detect possible common methodological biases. The results substantiated that there were 12 factors with characteristic values greater than 1, and the amount of variance explained by the first factor was 26.6%, which was less than the critical criterion of 40.0%. Thus, the influence of standard methodological deviations was excluded from this study.

### Demographic variables

**Fig. 2:**
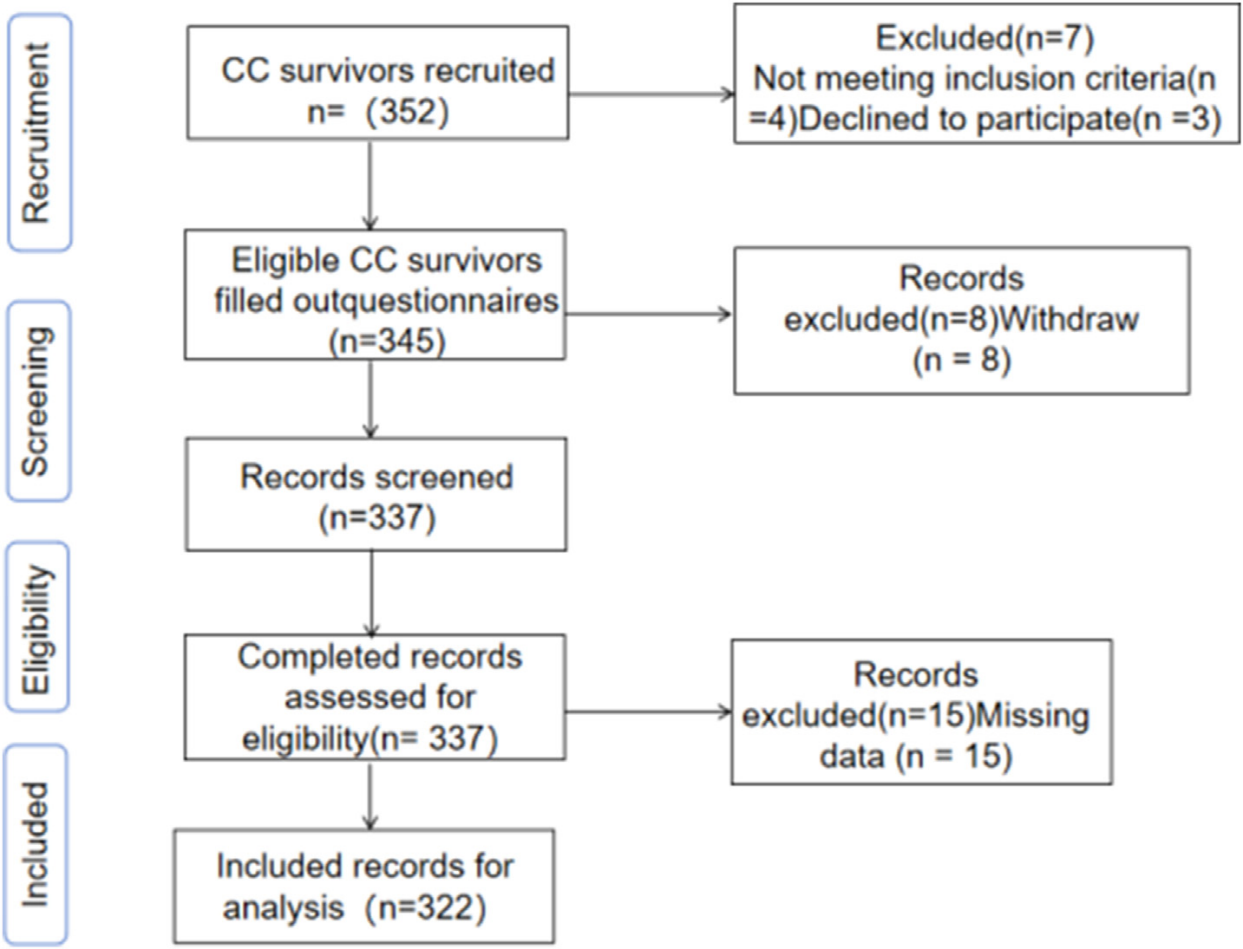
Flow diagram of study recruitment process. CC, cervical cancer.

A total of 352 patients with cervical cancer were recruited, of whom 345 met the inclusion criteria (98.0%). Twenty-three participants were excluded due to dropout or incomplete responses. Ultimately, 322 valid questionnaires were collected for final analysis, yielding a valid recovery rate of 91.5% (Fig. 2). As shown in Table 1, the participants' average age was 50.25 ​± ​11.75 years, ranging from 19 to 74 years; Additional information is presented in Table 1.

**Table 1:** Demographic and disease-related characteristics of the sample *(N* ​= ​322).

Characteristics	*n*	%
**Age (years)**		
18-39	83	25.8
40-59	168	52.2
60-79	71	22.0
**Education level**		
Junior high school or below	103	32.0
High school or secondary vocational	106	32.9
College or above	113	35.1
**Monthly income (yuan)**		
3000–6999	34	10.6
7000–10,000	110	34.2
> 10,000	178	55.3
**Occupation**		
Employed	127	39.4
Unemployed	70	21.7
Other	125	38.8
**Marital status**		
Married	286	88.8
Unmarried	10	3.1
Divorced or widowed	26	8.1
**Comorbidities**		
Yes	80	24.8
No	242	75.2
**Patient delay**		
Yes	120	37.3
No	202	62.7
**Initial symptoms**		
Abnormal bleeding	176	54.7
Contact bleeding	55	17.1
Abnormal vaginal discharge	26	8.1
Lumbar abdominal pain	14	4.3
Physical examination	46	14.3
Other	5	1.6
**Stage of cancer**		
Precancerous condition	49	15.2
I	62	19.3
II	102	31.7
III	88	27.3
IV	21	6.5

### Correlation analyses among the investigated variables

As presented in Table 2, the mean scores for perceived social support, health literacy, stigma, and perceived barriers to health care decision-making were 56.51 ​± ​7.16, 81.27 ​± ​12.41, 48.90 ​± ​11.37, and 36.45 ​± ​9.47, respectively. Perceived social support and perceived barriers to health care decision-making were categorized as moderate, while stigma was classified as moderate to high. Pearson correlation analysis revealed a moderate positive correlation between health literacy and perceived social support (*r* ​= ​0.41, *P* ​< ​0.001), indicating that higher health literacy is associated with better perceived social support. A moderate negative correlation was observed between health literacy and stigma (*r* ​= ​−0.42, *P* ​< ​0.001), suggesting that increased health literacy is associated with reduced stigma. A significant negative correlation was found between perceived social support and stigma (*r* ​= ​−0.64, *P* ​< ​0.001), suggesting that higher levels of social support are linked to lower levels of stigma. Furthermore, stigma was strongly positively correlated with health care decision-making barriers (*r* ​= ​0.61, *P* ​< ​0.001), indicating that higher stigma is associated with greater more significant to decision-making. Health literacy also showed a moderate negative correlation with health care decision-making barriers (*r* ​= ​−0.40, *P* ​< ​0.001), implying that higher health literacy is associated with fewer perceived barriers to health care decision-making.

**Table 2:** Descriptive satatistics and correlation between health literacy, perceived social support, stigma, and barriers to health care-seeking decision.

Variables	Mean ​± ​SD	Health literacy	Perceived social support	Stigma
Health literacy	81.27 ​± ​12.41			
Perceived social support	56.51 ​± ​7.16	0.41∗		
Stigma	48.90 ​± ​11.37	−0.42∗	−0.64∗	
Perceived barriers to health care-seeking decision	36.45 ​± ​9.47	−0.40∗	−0.50∗	0.61∗

SD, standard deviation. ∗*P* ​< ​0.01.

### The mediating effect of stigma and perceived social support

**Fig. 3:**
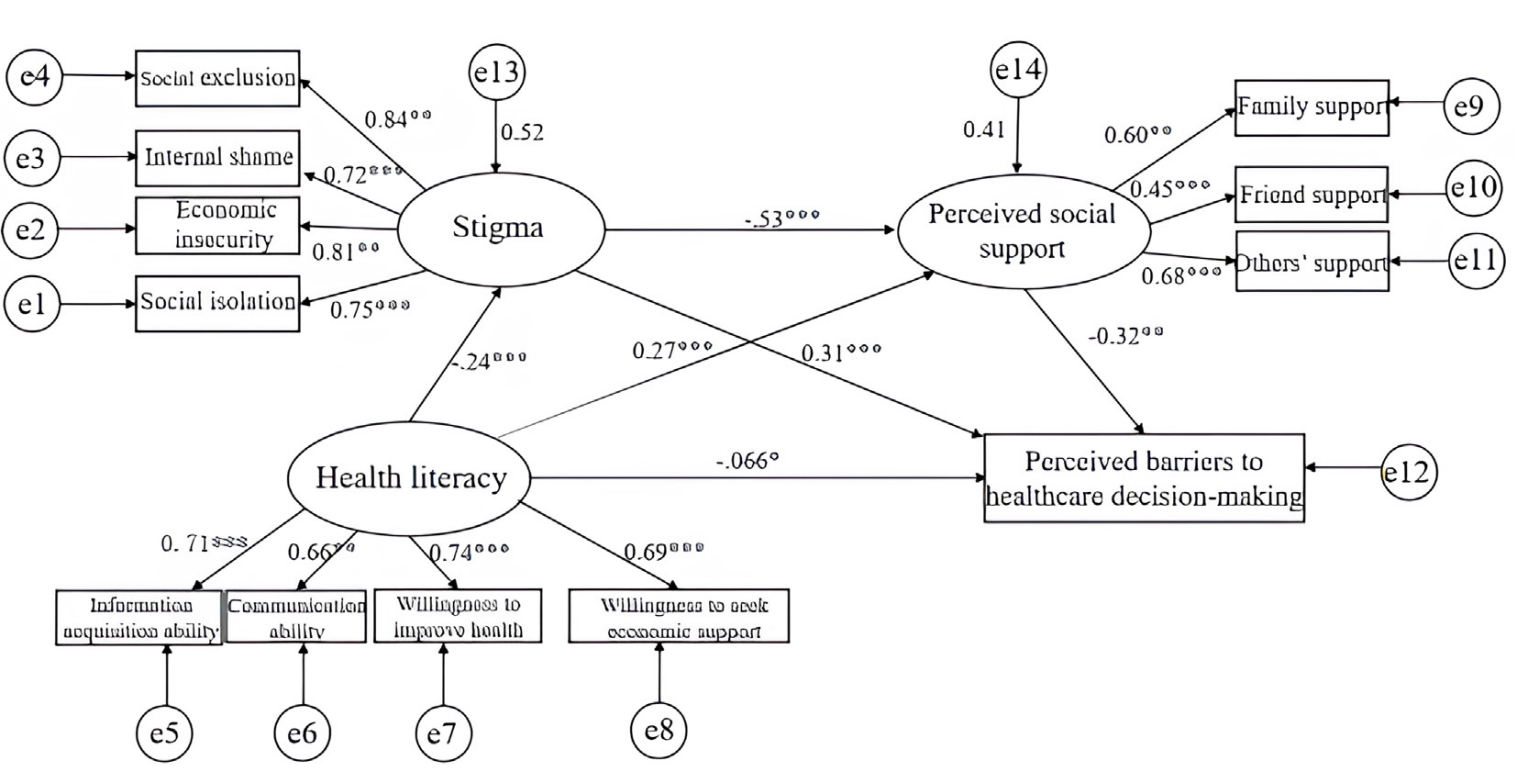
Mediation effect model.

A structural equation model was constructed using Amos, with health care decision-making barriers as the dependent variable, health literacy as the independent variable, and perceived social support and stigma as mediating variables (​Fig. 3). The model was estimated using the maximum likelihood method. Based on modification indices greater than 3, non-significant paths were removed and correlations between residuals were added. The model was then re-estimated and showed a good fit to the data.

The results of the bootstrap analysis show that the total effect of health literacy on health care decision-making barriers is −0.267, with a 95% confidence interval (CI) of [−0.334, −0.199], which excludes 0, indicating statistical significance. The direct effect of health literacy on health care decision-making barriers is −0.066 (95% CI: [−0.130, −0.003]), which is also statistically significant and suggests a negative influence. For the indirect effects, the total effect for the three indirect paths is −0.200 (95% CI: [−0.260, −0.151]), indicating that health literacy negatively affects decision-making barriers through stigma and perceived social support. These findings collectively support the mediating roles of stigma and perceived social support in the relationship between health literacy and health care decision-making barriers, thereby validating our hypotheses (Table 3).

**Table 3:** Mediation effect Bootstrap test and effect sizes.

Paths	Effect size	SE	95% CI	*P*	Proportion of total effect
Path 1	−0.073	0.016	−0.109, −0.044	< 0.001	27.5%
Path 2	−0.086	0.023	−0.133, −0.045	< 0.001	32.3%
Path 3	−0.041	0.011	−0.067, −0.021	< 0.001	15.4%
Total indirect effect	−0.200	0.028	−0.260, −0.151	< 0.001	75.1%
Direct effect	−0.066	0.032	−0.130, −0.003	0.041	24.9%
Total effect	−0.267	0.034	−0.334, −0.199	< 0.001	100.0%

SE, standard error; CI, confidence interval. Path 1: health literacy →stigma→health care decision-making barriers; Path 2: health literacy→perceived social support→health care decision-making Barriers; Path 3: health literacy →stigma → perceived social support→health care decision-making barriers.

## Discussion

While previous studies have primarily examined the direct relationship between health literacy and health care decision-making capabilities in patients with cervical cancer, the psycho-social mechanisms underlying this relationship remain under-explored.^28^ The psycho-social mechanisms underlying this relationship remain under-explored. Unlike the single-mediator model proposed by Mackert,^29^ our study identifies two distinct pathways through which health literacy impacts decision-making barriers. Firstly, health literacy directly influences decision-making obstacles by reducing cognitive biases. Secondly, it exerts an indirect effect by mitigating stigma and enhancing the utilization of social support. The sequential mediation aligns with the 10.13039/100018696Health Belief Model's theoretical framework,^18^ which posits that cognitive factors (health literacy) interact with emotional barriers (stigma) and environmental resources (social support) to shape health behaviors.

Relationship between health literacy and barriers to health care Decision-Making.

Our study found a significant negative correlation between health literacy and health care decision-making barriers, with patients having higher health literacy encountering fewer obstacles. This is consistent with prior studies that state that better health literacy leads to wiser and more timely decisions. Mackert's research^29^ demonstrated that women with higher health literacy are better at understanding complex medical information, thereby reducing fears and misunderstandings about treatment and overcoming systemic health care barriers. For patients with cervical cancer, higher health literacy enables them to understand their treatment options better, alleviate fears associated with uncertainty, and make more timely and informed health care decisions.

However, while improved health literacy generally supports better decision-making, some studies have found that other factors,^30^^,^^31^ such as emotional and social pressures, may still pose significant barriers. Salehiniya^18^ found that although patients with higher health literacy have a clearer understanding of treatment options, they still face substantial emotional and social pressures during the decision-making process, which, in some cases, can delay timely health care decisions. These non-cognitive factors may lead patients with higher health literacy to delay or avoid health care decisions in certain situations. Therefore, in addition to improving health literacy, addressing emotional support and social-environmental factors is equally crucial in facilitating timely decision-making. Improving patients' health literacy is key, and it is essential to implement a range of intervention strategiesBy developing easy-to-understand educational resources (such as graphics and videos), organizing health lectures, and personal counselling,^32^ measures can be taken to help patients understand treatment plans and alleviate concerns.^33^ These interventions can significantly improve patients' health literacy and decision-making ability, enable them to actively participate in the treatment process and make wise choices.

The mediating role of stigma between health literacy and health care decision-making barriers.

This study confirms Hypothesis 2, indicating that stigma plays a mediating role in the relationship between health literacy and health care decision-making barriers. This aligns with Corrigan's findings^34^ that stigma hinders individuals from seeking medical help and participating in mental health care. Existing literature suggests^35^ that cancer-related stigma exacerbates patients' hesitation and avoidance of medical care, leading to delays in treatment and poorer health outcomes. Moreover, Chan^36^ found that cancer patients experiencing high levels of societal stigma are more likely to delay decision-making due to emotional distress and fear of societal judgment, further complicating their treatment choices and adherence to medical advice.

Our study further reveals that stigma not only directly impacts health care decision-making but also indirectly influences it through health literacy. Specifically, the emotional burden of stigma can reduce patients' willingness to seek information or engage in treatment decisions, even if they have the cognitive ability to understand complex medical information.^37^ This is particularly relevant for patients with cervical cancer, who may feel marginalized or stigmatized due to the nature of their condition. The presence of stigma diminishes their confidence in making wise decisions, thereby exacerbating decision-making barriers.^38^ This mechanism is consistent with Carter's^39^ findings, which indicate that even patients with higher health literacy are less likely to actively engage in health care decision-making when they experience a higher level of stigma. Research has shown that psychological counselling and social support can effectively reduce the stigma of cancer patients and improve treatment adherence and quality of life.^40^ Specific interventions include regular psychological counselling, establishing specialized support groups, and encouraging patients to join peer support networks.^41^ These interventions help reduce stigma, promote self-acceptance, and support positive attitudes towards treatment.

The mediating role of perceived social support between health literacy and health care decision-making barriers.

The mediation model analysis shows that perceived social support plays a significant mediating role in the relationship between health literacy and perceived barriers to health care decision-making (Hypothesis 3 is supported). Specifically, patients who perceive stronger social support encounter fewer barriers when making health care decisions.^42^ This finding is consistent with existing literature, emphasizing the crucial buffering role of social support in patients' health care decision-making processes. For instance, Nazione's study^43^ analyzed the verbal social support strategies provided by surgeons and peers to patients with breast cancer and found that surgeons mainly provide informational support. In contrast, patients with peers receive additional network-based support from surgeons. This suggests that social support plays an important role in enhancing patients' confidence and coping ability in decision-making. Furthermore, Widén's research^44^ indicates that individuals with higher information literacy tend to perceive more social support, enable them to access and utilize information more effectively, thereby gaining broader resources and support. These findings further emphasize the interaction between information literacy and social support, as well as their positive impact on the decision-making process.

Our study further reveals that patients with cervical cancer who lack social support face significantly more significant obstacles in health care decision-making. Research on patients with breast cancer shows that emotional and practical support from family and friends can effectively alleviate confusion and uncertainty in the treatment process, thus promoting more active participation in health care decision-making.^45^ Our results align with these findings, indicating that a strong social support network not only reduces decision-making barriers but also significantly improves the quality of health decisions.^46^ To enhance social support, health care institutions can facilitate patients to join relevant support groups, such as cancer patient groups or online communities. These platforms not only provide emotional support but also give patients the opportunity to share treatment experiences and access more medical resources and information.^47^ The support of family members and friends is also crucial for patients' treatment decisions. Research shows that family and social support play a role in emotional regulation and treatment adherence.^40^

Chain-mediated role of stigma and perceived social support between health literacy and health care decision-making barriers.

This study is the first to reveal the chain-mediated effect of stigma and perceived social support on the relationship between health literacy and health care decision-making barriers. Our research findings indicate that health literacy indirectly improves patients' health care decision-making abilities by reducing stigma and enhancing perceived social support. Specifically, higher health literacy effectively alleviates the negative emotions induced by cancer-related stigma, thereby reducing decision-making barriers. Additionally, perceived social support may help patients overcome decision-making challenges by providing emotional and informational support, thereby enhancing their confidence and motivation in treatment decisions.

Similarly, our findings are consistent with Khok's study,^48^ which emphasized that cancer patients often experience decision delays and health care avoidance due to societal stigma. Our research further reveals that improving health literacy not only alleviates stigma but also reduces decision-making delays and avoidance behaviors by enhancing social support. Specifically, higher health literacy effectively reduces the negative emotions associated with social stigma, thereby reducing delays and avoidance in health care decision-making. This empowers patients to engage more actively in medical decision-making, thereby improving decision quality and fostering more positive decision-making behaviors.

Furthermore, we confirmed the crucial role of perceived social support in reducing health care decision-making barriers. Our study found that perceived social support significantly reduced the barriers that patients encountered in the decision-making process. This is in line with Kim's research,^49^ which found that social support positively affects the emotional health of women with breast cancer, such as reducing depressive symptoms and other negative emotions and helping them make more positive health care decisions. Our findings further confirm the critical role of perceived social support in alleviating stigma and decision-making barriers, emphasizing its vital influence on the health care decision-making process.

Our study further elucidates the interaction among health literacy, stigma, and social support, indicating that these factors collectively influence the quality of patients' health care decisions. These findings provide valuable theoretical insights for the development of more effective intervention strategies.

It is worth noting that the chain-mediating pathway of:stigma→perceived social support plays a crucial bridging role between health literacy and health care decision-making barriers. Therefore, the results of this study expand our understanding of the key factors influencing health care decision-making among patients with cervical cancer and clarify the mechanisms for enhancing decision-making capacity. The results indicate that higher levels of psychological support, health education, and social support networks are associated with fewer health care decision-making barriers and improved health outcomes in cervical cancer patients. However, given the cross-sectional design of the study, no causal inferences can be drawn. Longitudinal studies are required to investigate the long-term effects of these factors further. These findings offer both a theoretical foundation and practical guidance for developing targeted clinical intervention strategies.

Additionally, when considering the chain-mediated effects of stigma and perceived social support, health care providers should implement integrated interventions targeting the following pathway: reduce stigma→enhance health literacy→strengthen perceived social support→improve decision-making capacity. Compared to interventions focusing on a single factor,^50^ an integrated approach involving psychological support, health education, and social support networks appears to be associated with reduced health care decision-making barriers among patients with cervical cancer. Further research, including longitudinal studies, is required to evaluate the long-term effects of these integrated approaches. By simultaneously addressing psychological and social factors, health care professionals can create a more supportive environment, enhance patients' informed decision-making ability, and encourage them to engage more actively in treatment plans.

### Implications for nursing practice and research

These findings suggest that nurses should play a proactive role in identifying stigma, providing tailored education, and fostering social support systems to reduce health care decision-making barriers in patients with cervical cancer. Future research should focus on longitudinal and interventional studies to evaluate the long-term effects of multifactorial strategies and explore additional influencing factors, thereby providing a theoretical basis for individualized nursing care.

### Limitations

There are several limitations to this study. Firstly, the sample was collected from only three Beijing tertiary hospitals through convenience sampling, which may lead to selection bias and restrict the generalizability of the findings. Future research should use more random and multi-site sampling, including rural and small/medium-sized city hospitals. Secondly, the cross-sectional design does not prove causality. Longitudinal studies are needed to clarify the –causal relationships between variables such as health literacy and stigma. Thirdly, relying on self-reported data may lead to reporting bias. Objective measures should be incorporated in future studies to improve accuracy. Finally, this study only considered stigma and perceived social support as mediators. Other factors such as cultural beliefs and personality traits may also be significant. A more comprehensive study is required to fully understand the relationship between health literacy and health care decision-making barriers among patients with cervical cancer.

## Conclusions

Overall, this study is the first to reveal that stigma negatively impacts health care decision-making in patients with cervical cancer and highlights the sequential mediating effect of health literacy and perceived social support. The chain-mediating effect of health literacy and social support provides significant practical implications for improving health care decision-making for patients with cervical cancer. Further research should be conducted in the future to improve and optimize interventions for patients with cervical cancer and enhance their health care decision-making ability and health outcomes.

## CRediT authorship contribution statement

**Hanjiao Kong**: Methodology, Software, Data curation, Data analysis, Writing Original draft preparation; **Jingzhi Geng**: Research conceptualization, Investigation; **Qiuhui Wang**: Validation, Data duration; **jie Zhu**: Data curation; **Hong Zhao**: Supervision, Writing – Reviewing and Editing. All authors have read and approved the final manuscript.

## Ethics statement

This study was approved by the Research Ethics Committee of Peking Union Medical College (Approval No. PUMCSON-2023-14) and was conducted in accordance with the 1964 Helsinki Declaration and its later amendments or comparable ethical standards. All participants provided written informed consent.

## Data availability statement

The data that support the findings of this study are available from the corresponding author, ND, upon reasonable request.

## Declaration of generative AI and AI-assisted technologies in the writing process

No AI tools/services were used during the preparation of this manuscript.

## Funding

This study received no external funding.

## Declaration of competing interest

The authors declare no conflict of interest.

## References

[1] Abu-Rustum N.R., Yashar C.M., Arend R. (2023). NCCN Guidelines® insights: cervical cancer, version 1.2024. J Natl Compr Cancer Netw.

[2] Perkins R.B., Wentzensen N., Guido R.S. (2023). Cervical cancer screening: a review. JAMA.

[3] Allahqoli L., Dehdari T., Rahmani A. (2022). Delayed cervical cancer diagnosis: a systematic review. Eur Rev Med Pharmacol Sci.

[4] Shimels T., Gashaw B., Gedif T. (2022). Association between delayed initiation of treatment indications and survival in patients with cervical cancer: a systematic review and meta-analysis protocol. PLoS One.

[5] Ouasmani F., Hanchi Z., Haddou Rahou B., Bekkali R., Ahid S., Mesfioui A. (2016). Determinants of patient delay in seeking diagnosis and treatment among Moroccan women with cervical cancer. Obstet Gynecol Int.

[6] Brand N.R., Ql C.A. (2019). Delays and barriers to cancer care in low- and middle-income countries: a systematic review. Oncologist.

[7] Omran S., Ismail A.A. (2010). Knowledge and beliefs of Jordanians toward colorectal cancer screening. Cancer Nurs.

[8] Ma J., Luo Y., Yang S. (2023). Patient delay and related influencing factors in Chinese women under 35 years diagnosed with cervical cancer: a cross-sectional study. Asia Pac J Oncol Nurs.

[9] Somanna S.N., Murthy S.N., Chaluvarayaswamy R. (2020). Time from self-detection of symptoms to seeking definitive care among cervical cancer patients. Asian Pac J Cancer Prev.

[10] Hasannejadasl H., Roumen C., Smit Y. (2022). Health literacy and eHealth: challenges and strategies. JCO Clin Cancer Inform.

[11] Nutbeam D., Lloyd J.E. (2021). Understanding and responding to health literacy as a social determinant of health. Annu Rev Publ Health.

[12] Ryman C., Warnicke C., Hugosson S. (2024). Health literacy in cancer care: a systematic review. Eur J Oncol Nurs.

[13] Behnamfar F.A.,M. (2015). Factors associated with delayed diagnosis of cervical cancer in Iran- A survey in Isfahan city. Asian Pac J Cancer Prev.

[14] Zewdie A., Shitu S., Kebede N. (2023). Determinants of late-stage cervical cancer presentation in Ethiopia: a systematic review and meta-analysis. BMC Cancer.

[15] Paneru B., Karmacharya A., Bharati A. (2023). Association between cancer stigma and cervical cancer screening uptake among women of Dhulikhel and Banepa, Nepal. PLoS One.

[16] Shariati-Sarcheshme M., Mahdizdeh M., Tehrani H. (2024). Women's perception of barriers and facilitators of cervical cancer Pap smear screening: a qualitative study. BMJ Open.

[17] Birhanu Z., Abdissa A., Belachew T. (2012). Health seeking behavior for cervical cancer in Ethiopia: a qualitative study. Int J Equity Health.

[18] Salehiniya H., Momenimovahed Z., Allahqoli L., Momenimovahed S., Alkatout I. (2021). Factors related to cervical cancer screening among Asian women. Eur Rev Med Pharmacol Sci.

[19] Stice E., Ragan J., Randall P. (2004). Prospective relations between social support and depression: differential direction of effects for parent and peer support?. J Abnorm Psychol.

[20] Adebola A., Adaeze A., Adeyimika D. (2024). Experiences and challenges of African American and sub-saharan African immigrant black women in completing pap screening: a mixed methods study. J Racial Ethn Health Dispar.

[21] Coleman D., Hurtado-de-Mendoza A., Montero A. (2024). Stigma, social support, and spirituality: associations with symptoms among Black, Latina, and Chinese American cervical cancer survivors. J Cancer Surviv.

[22] Tsang S., Royse C.F., Terkawi A.S. (2017). Guidelines for developing, translating, and validating a questionnaire in perioperative and pain medicine. Saudi J Anaesth.

[23] Jordan J.E., Osborne R.H., Buchbinder R. (2011). Critical appraisal of health literacy indices revealed variable underlying constructs, narrow content and psychometric weaknesses. J Clin Epidemiol.

[24] Al-Hassan M.A., Omran S.M. (2005). The effects of health beliefs on health care-seeking decisions of Jordanian patients with myocardial infarction symptoms. Int J Nurs Pract.

[25] Li P.W., Lee D.T., Yu D.S. (2014). Psychometric evaluation of the perceived barriers to health care-seeking decision in Chinese patients with acute coronary syndromes. Heart Lung.

[26] Fife B.L., Wright E.R. (2000). The dimensionality of stigma: a comparison of its impact on the self of persons with HIV/AIDS and cancer. J Health Soc Behav.

[27] Zimet G.D., Powell S.S., Farley G.K. (1990). Psychometric characteristics of the multidimensional scale of perceived social support. J Pers Assess.

[28] van der Gaag M., Heijmans M., Spoiala C. (2022). The importance of health literacy for self-management: a scoping review of reviews. Chron Illness.

[29] Mackert M., Mabry-Flynn A.E., Donovan E.E. (2019). Health literacy and perceptions of stigma. J Health Commun.

[30] Xiang Y., Jiang H., Zhao L., Liu Q., Lin H. (2022). Delays in seeking medical services in elderly patients with senile cataract. Front Psychol.

[31] Pati S., Chauhan A.S., Mahapatra S., Nayak S., Nayak S., Weller D. (2017). Treatment experiences of women with reproductive cancers in Odisha, India: a qualitative exploration of enablers and barriers. Asian Pac J Cancer Prev.

[32] Stormacq C., Wosinski J., Boillat E. (2020). Effects of health literacy interventions on health-related outcomes in socioeconomically disadvantaged adults living in the community: a systematic review. JBI Evid Synth.

[33] Glaser J., Nouri S., Fernandez A. (2020). Interventions to improve patient comprehension in informed consent for medical and surgical procedures: an updated systematic review. Med Decis Mak.

[34] Corrigan P.W., Druss B.G., Perlick D.A. (2014). The impact of mental illness stigma on seeking and participating in mental health care. Psychol Sci Publ Interest.

[35] Johnson S.E., Samson M. (2024). Cancer stigma: the need for policy and programmatic action. J Natl Cancer Inst Monogr.

[36] Chen C.P., Kung P.T., Wang Y.H. (2019). Effect of time interval from diagnosis to treatment for cervical cancer on survival: a nationwide cohort study. PLoS One.

[37] Wu J., Zeng N., Wang L., Yao L. (2023). The stigma in patients with breast cancer: a concept analysis. Asia Pac J Oncol Nurs.

[38] Nyblade L., Stockton M., Travasso S. (2017). A qualitative exploration of cervical and breast cancer stigma in Karnataka, India. BMC Womens Health.

[39] Carter-Harris L. (2015). Lung cancer stigma as a barrier to medical help-seeking behavior: practice implications. J Am Assoc Nurse Pract.

[40] Rastad C., Martin C., Asenlof P. (2014). Barriers, benefits, and strategies for physical activity in patients with schizophrenia. Phys Ther.

[41] Seidman A.J., Wade N.G., Geller J. (2022). The effects of group counseling and self-affirmation on stigma and group relationship development: a replication and extension. J Counsel Psychol.

[42] Yang B., Lu W., Xuan Y., Hao C., Huang X. (2025). The influences of social support expressed from doctors and disclosed from peers on patient decision-making: an analysis from the online health community. Sci Rep.

[43] Nazione S., Silk K.J., Robinson J. (2016). Verbal social support for newly diagnosed breast cancer patients during surgical decision-making visits. J Commun Healthc.

[44] Widén G., Ahmad F., Huvila I. (2023). Connecting information literacy and social capital to better utilise knowledge resources in the workplace. J Inf Sci.

[45] Li J., Yuan B., Wang Y. (2021). Research progress in decision-making aids for breast cancer patients. Zhong Nan Da Xue Xue Bao Yi Xue Ban.

[46] Li Q., Liu L., Gu Z., Li M., Liu C., Wu H. (2023). Sense of coherence mediates perceived social support and depressive and anxiety symptoms in cervical cancer patients: a cross-sectional study. BMC Psychiatry.

[47] Guan M., Han J.Y., Shah D.V., Gustafson D.H. (2021). Exploring the role of social support in promoting patient participation in health care among women with breast cancer. Health Commun.

[48] Khok X.W., Ng W., Lee A.Y. (2024). Journey towards resiliency: a systematic review and meta-synthesis of cancer patients' experiences. Eur J Oncol Nurs.

[49] Kim J., Han J.Y., Shaw B. (2010). The roles of social support and coping strategies in predicting breast cancer patients' emotional well-being: testing mediation and moderation models. J Health Psychol.

[50] Dhakal K., Chen C., Wang P., Mboineki J.F., Adhikari B. (2024). Existing psychological supportive care interventions for cervical cancer patients: a systematic review and meta-analysis. BMC Public Health.

